# ﻿A new species of *Lumbrineriopsis* (Annelida, Eunicida, Lumbrineridae) from southeastern Brazil

**DOI:** 10.3897/zookeys.1174.101059

**Published:** 2023-08-09

**Authors:** Nálita Maria Scamparle Teodoro, Tatiana Menchini Steiner, Antonia Cecília Zacagnini Amaral

**Affiliations:** 1 Pós-graduação em Biologia Animal, Departamento de Biologia Animal, Instituto de Biologia, Universidade Estadual de Campinas, Rua Monteiro Lobato, 255, 13083-862, Campinas, São Paulo, Brazil Universidade Estadual de Campinas Campinas Brazil; 2 Laboratório de Biodiversidade Bêntica Marinha, Departamento de Biologia Animal, Instituto de Biologia, Universidade Estadual de Campinas, Rua Monteiro Lobato, 255, 13083-62, Campinas, São Paulo, Brazil Universidade Estadual de Campinas Campinas Brazil

**Keywords:** *Lumbrineriopsisdulcis* sp. nov., morphology, new genus record, new species, polychaetes, South Atlantic, taxonomy

## Abstract

*Lumbrineriopsisdulcis***sp. nov.** is morphologically described from the continental shelf and slope of Espírito Santo and the Campos Basin of Rio de Janeiro state, southeastern Brazil, at depths between 14 and 400 m. *Lumbrineriopsismucronata* is the only species of the genus recorded until now in Brazil. The new species differs from other congeneric species in its jaw-apparatus morphology with unfused mandibles and a fixed number of simple limbate chaetae and simple, bidentate, hooded hooks in each parapodium. This paper aims to fill the gap in knowledge on the family Lumbrineridae, which has not been studied in Brazil for the last 25 years and provides the first record of the genus from Espírito Santo and Rio de Janeiro states. This record is significant given the damage to the marine ecosystem of the Espírito Santo region due to the 2015 rupture of the Samarco mining company dam, the largest environmental disaster in Brazil’s history. In addition, this region has important environmental conservation units such as Costa das Algas Environmental Protection Area, Santa Cruz Wildlife Refuge, and Comboios Biological Reserve. All these preserved areas are of paramount importance for the protection of marine biological diversity.

## ﻿Introduction

The family Lumbrineridae Schmarda, 1861 is composed of species with similar external morphology. Their identification was based, until 1990, on characters of the prostomium and parapodial lobes, as well as the type of chaetae. Consequently, the classification resulted in a generic system that arranged the existing species into three or four genera (Hartman 1944; Fauchald 1970; Zanol et al. 2021), which lead to descriptions with insufficient information. In addition, jaw structure was little explored. Studies by Orensanz (1973, 1990) proposed new morphological characters, especially related to jaw apparatus, representing an advancement of the taxonomy of the family and promoting the description of new genera and species (Orensanz 1990; Carrera-Parra 2001; Carrera-Parra and Orensanz 2002; Carrera-Parra 2004; Carrera-Parra 2006; Katsiaras et al. 2017).

Lumbrineridae comprises about 279 valid species in 19 genera (Carrera-Parra 2006; Zanol et al. 2021). In Brazil, 32 species have been recorded belonging to the genera *Augeneria*, *Cenogenus*, *Lumbricalus*, *Lumbrinerides*, *Lumbrineriopsis*, *Lumbrineris*, *Lysarete*, *Ninoe*, and *Scoletoma*; they occur from the intertidal zone to the continental shelf and slope, reaching 1126 m deep, on both consolidated and unconsolidated substrates, as well as associated with algae, sponges, and corals (Costa et al. 2017; Amaral et al. 2023). However, only Camargo and Lana (1995a, 1995b) have carried out more comprehensive taxonomic studies of the family and described 17 species from southern and southeastern coasts of Brazil, including two new species, *Scoletomamainae* and *Scoletomacurtolobata*.

The genus *Lumbrineriopsis* was erected by Orensanz (1973) for a group very small, narrow and long-bodied lumbrinerids having simple, bidentate, hooded hooks, long and slender maxillary carriers, and maxillae IV with a fringe of denticles. Two species were initially known: *L.paradoxa* (Saint-Joseph, 1888) and *L.mucronata* (Ehlers, 1908). Subsequently, *L.tsushimaensis* Imajima & Higuchi, 1975, *L.gardineri* Miura, 1980, and *L.gasconiensis* Miura, 1980 were described. *Lumbrineriopsismucronata* has been recorded in Brazil where it occurs in silty-clayey and sandy-mud bottoms of bays, from 8 to 90 m deep, in the states of Rio de Janeiro and Santa Catarina (Camargo and Lana 1995a).

Herein, *Lumbrineriopsisdulcis* sp. nov. is described based on material from the states of Espírito Santo and Rio de Janeiro. The new species is grouped together with *L.mucronata* and *L.paradoxa*, whose distinctive feature is the presence of unfused jaws.

This is the first record of the genus from Espírito Santo state, which is significant given the damage to the marine ecosystem in this region due to the rupture of the dam belonging to the Samarco mining company. In 2015 the company dumped 50 million m^3^ of ore tailings into the Doce River, which flows into an estuary in the Atlantic Ocean. This was the biggest environmental disaster in the history of Brazil. In addition, this region has important environmental conservation units such as Costa das Algas Environmental Protection Area, Santa Cruz Wildlife Refuge, and Comboios Biological Reserve. All these preserved areas are of paramount importance for the protection of marine biological diversity.

## ﻿Material and method

The specimens were collected along southeastern Brazil during two major Brazilian oceanographic research projects carried out between 2008 and 2013 in soft bottoms from 12 to 3301 m depth and coordinated by CENPES/PETROBRAS (“Assessment of the environmental heterogeneity of the Campos Basin” (HABITATS) and “Marine environmental characterization of the Espírito Santo and northern portion of the Campos Basins” (AMBES)). The collected samples were fixed in 4% formaldehyde, and the specimens were preserved in 70% alcohol.

For detailed visualization of the jaw apparatus, some individuals were immersed in 0.2% sodium hypochlorite for 12 min and subsequently immersed in Hoyer’s solution. Chaetigers from anterior, median, and posterior regions, including parapodia, were dissected and immersed in Aquatex aqueous mounting medium. Individuals were examined using both Zeiss Stereo Discovery v. 2.0 and Zeiss Axioskop 2 Plus microscopes. Two specimens for scanning electron microscopy (SEM) imaging were dehydrated in a series of baths from 70% to 100% ethanol, then carried to the critical-point drying, metalized, and analyzed using a JEOL 6610 LV scanning electron microscope at the Laboratory of Electron Microscopy (LME), Instituto de Biologia, Universidade Estadual de Campinas (UNICAMP). The specimens are deposited at the Polychaeta Collection (ZUEC-POL) of the Museu de Diversidade Biológica (MDBio), Instituto de Biologia, UNICAMP and the Polychaeta Collection (MZUSP) at the Museu de Zoologia, Universidade de São Paulo (USP), Brazil.

Based on the morphology of mandibles (Figs 7, 8A–C) the specimens were divided into two groups: up to 0.29 mm wide (juvenile specimens) and between 0.3 and 0.9 mm (adults).

## ﻿Results

### ﻿Systematics

**Class Polychaeta Grube, 1850**

**Order Eunicida Dales, 1962**

**Family Lumbrineridae Schmarda, 1862**

#### ﻿Genus Lumbrineriopsis

Taxon classificationAnimaliaEunicidaLumbrineridae

Orensanz, 1973

948E98FA-8044-5B88-8B50-0EFBA7C82B52

##### Diagnosis.

Antennae and eyes absent. Notopodia small, without branchiae. Limbate chaetae and simple bidentate hooded hooks. Pygidium without anal cirri. Jaw apparatus with four pairs of maxillae, maxillary carriers longer than MI, joined to 1/2 of its base. MI forceps-like, without inner accessory teeth, with attachment lamella. MII with ligament, wide attachment lamella along 2/3 of posterior lateral edge; without connecting plates. MIII completely pigmented, narrow attachment lamella along 1/2 of posterior lateral edge. MIV completely pigmented, wide attachment lamella. Mandibles unfused, fused along the entire inner margin or up to 3/4 of its length (modified from Carrera-Parra 2006).

##### Type species.

*Lumbriconereismucronata* Ehlers, 1908.

#### ﻿ Lumbrineriopsis dulcis sp. nov.

Taxon classificationAnimaliaEunicidaLumbrineridae

F46F8A66-B9DC-5173-8ADB-148B23FEBE3A

https://zoobank.org/AA92A29E-6512-4F7C-AB79-D9422651C307

Figs 1 , 2 , 3 , 4 , 5 , 6 , 7 , 8 ; Table 1

##### Description.

**Figure 1.**
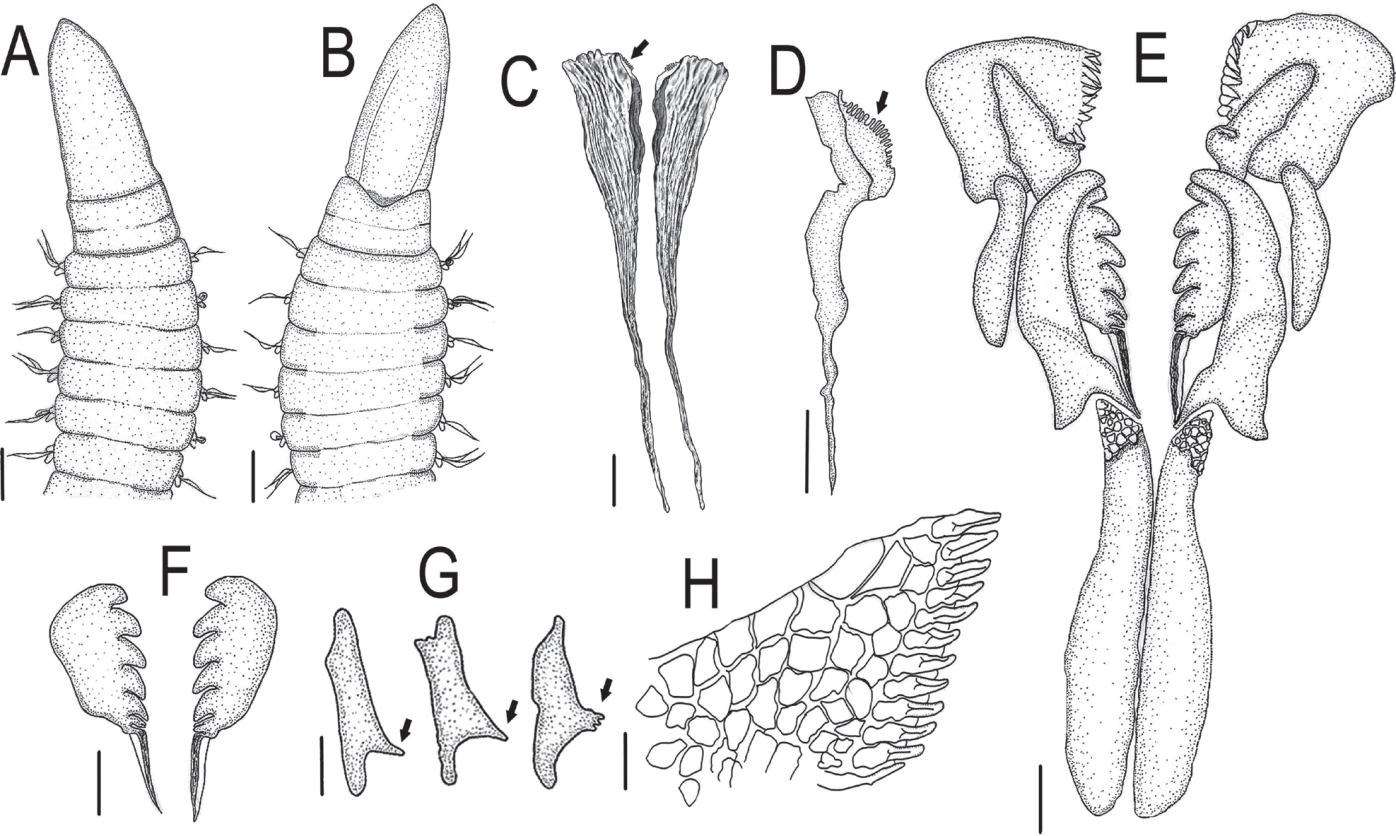
*Lumbrineriopsisdulcis* sp. nov. **A** anterior end, dorsal view (ZUEC-POL 21515) **B** anterior end, ventral view (ZUEC-POL 21515) **C** mandibles (adult), dorsal view (ZUEC-POL 22897) **D** right mandible (juvenile), ventral view (ZUEC-POL 22900) **E** jaw apparatus, dorsal view (ZUEC-POL 21524) **F** MII, detailed view (ZUEC-POL 21524) **G** MIII, detailed view (ZUEC-POL 21524) **H** MIV detailed view (ZUEC-POL 21524). Arrows of **C, D** teeth of mandibles; arrows of **G** teeth of MIII. Scale bars: 125 µm (**A, B**); 31.25 µm (**C**); 25 µm (**D**); 31.25 µm (**E–G**); 12.5 µm (**H**).

**Figure 2.**
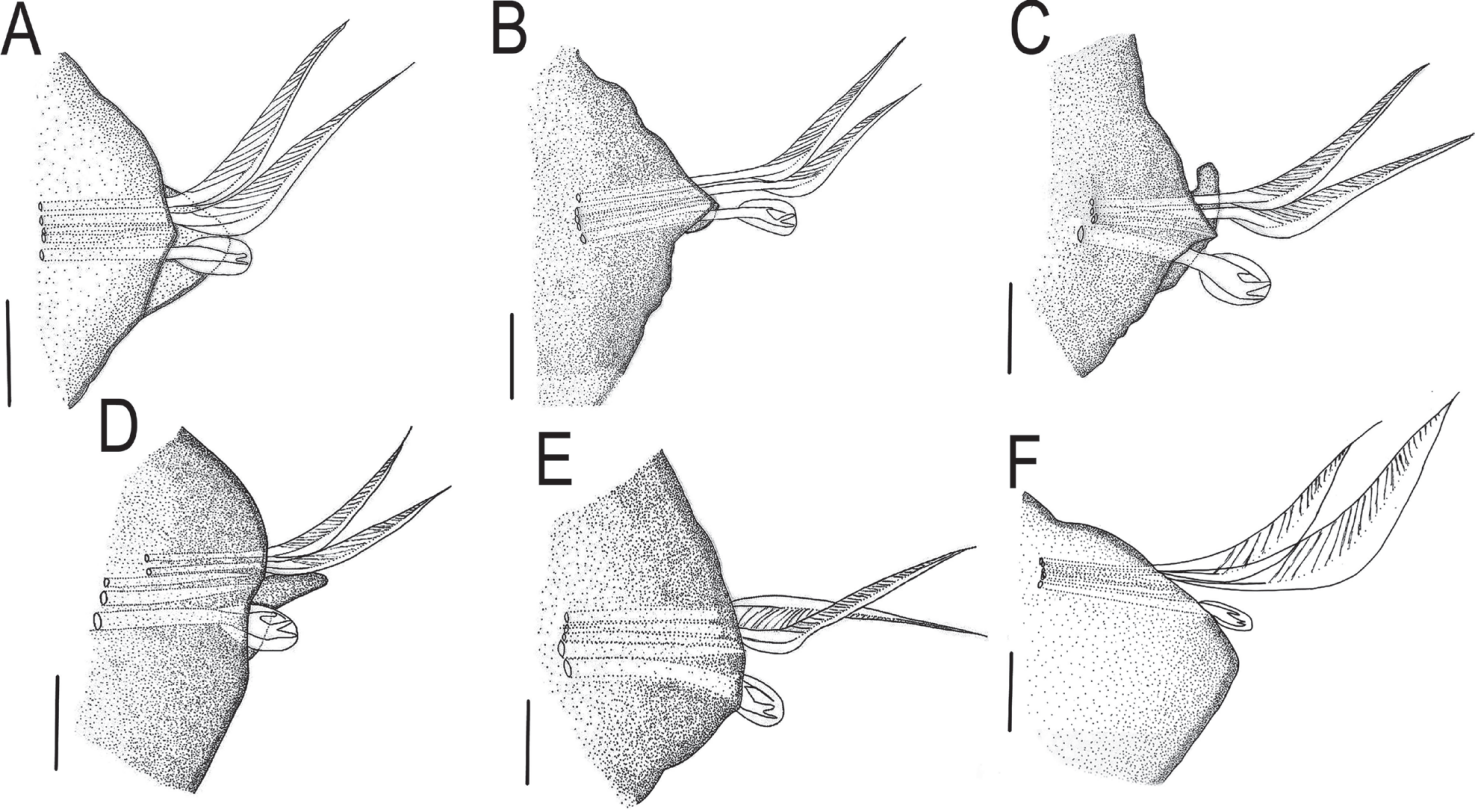
*Lumbrineriopsisdulcis* sp. nov. (ZUEC-POL 21527) **A** parapodium 1 **B** parapodium 5 **C** parapodium 11 **D** parapodium 59 **E** parapodium 97 **F** parapodium 100. All parapodia in anterior view. Scale bars: 31.25 µm.

**Figure 3.**
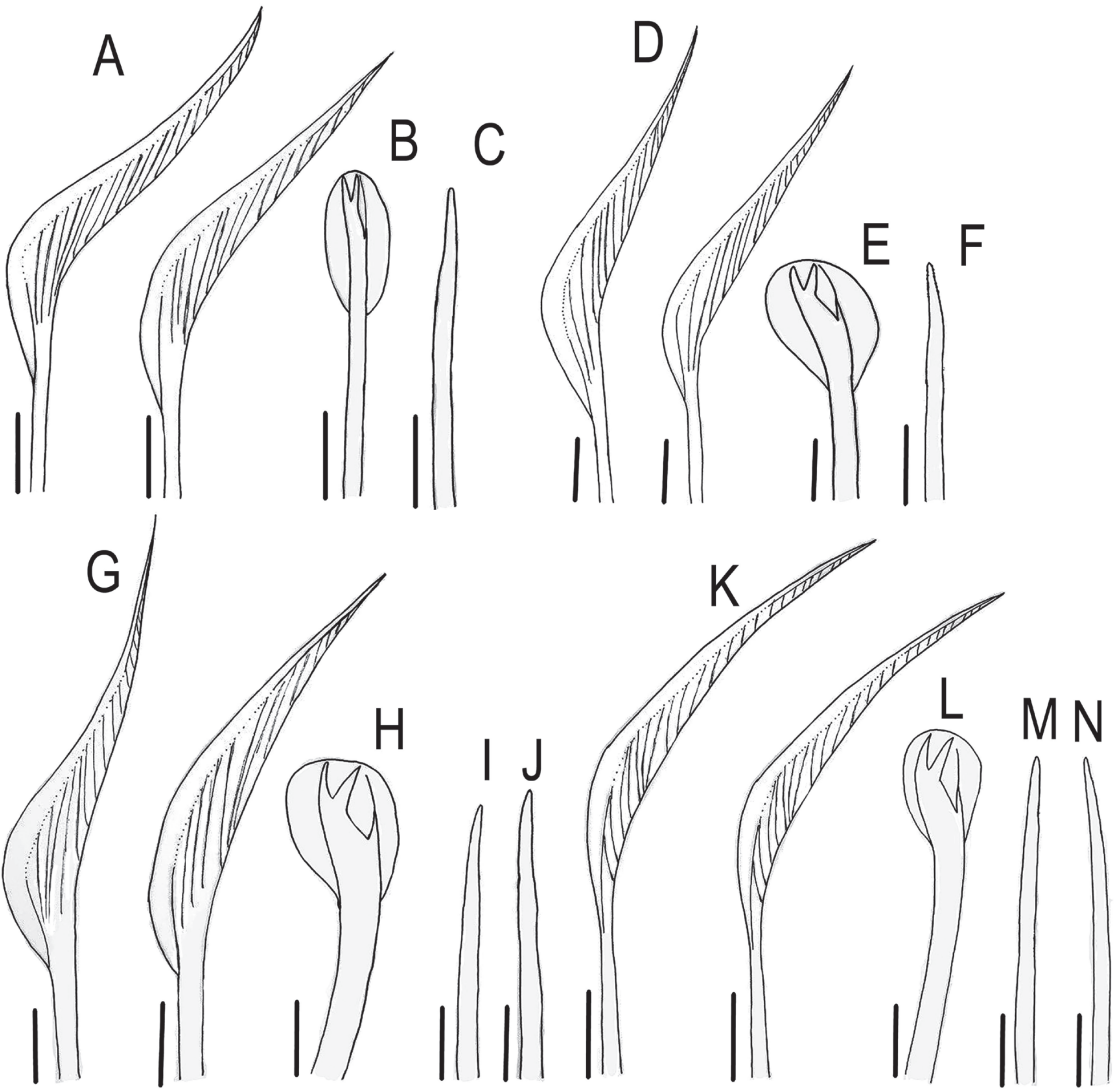
*Lumbrineriopsisdulcis* sp. nov. (ZUEC-POL 21527) **A** simple limbate chaetae **B** simple bidentate hooded hook **C** acicula **D** simple limbate chaetae **E** simple bidentate hooded hook **F** acicula **G** simple limbate chaetae **H** simple bidentate hooded hook **I, J** aciculae **K** simple limbate chaetae **L** simple bidentate hooded hook **M, N** aciculae **A–C** parapodium 1 **D–F** parapodium 5 **G–J** parapodium 11 **K–N** parapodium 59. Scale bars: 31.25 µm.

**Figure 4.**
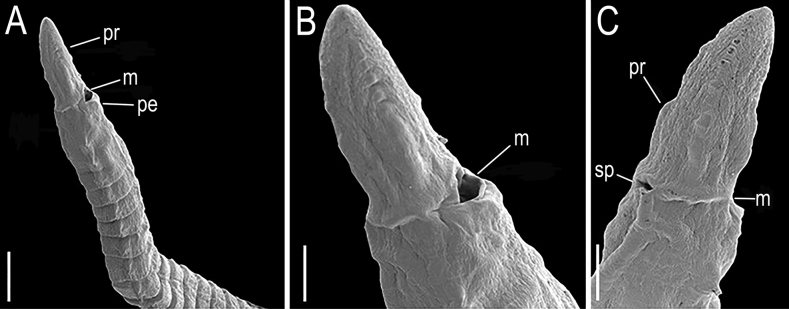
*Lumbrineriopsisdulcis* sp. nov. (SEM micrographs). (ZUEC-POL 21523) **A, B** anterior end, ventrolateral view **C** anterior end, dorsolateral view. **m** – mouth, **pe** –peristomium, **pr** – prostomium, **sp** – sensory pit. Scale bars: 200 µm (**A**); 100 µm (**B, C**).

**Figure 5.**
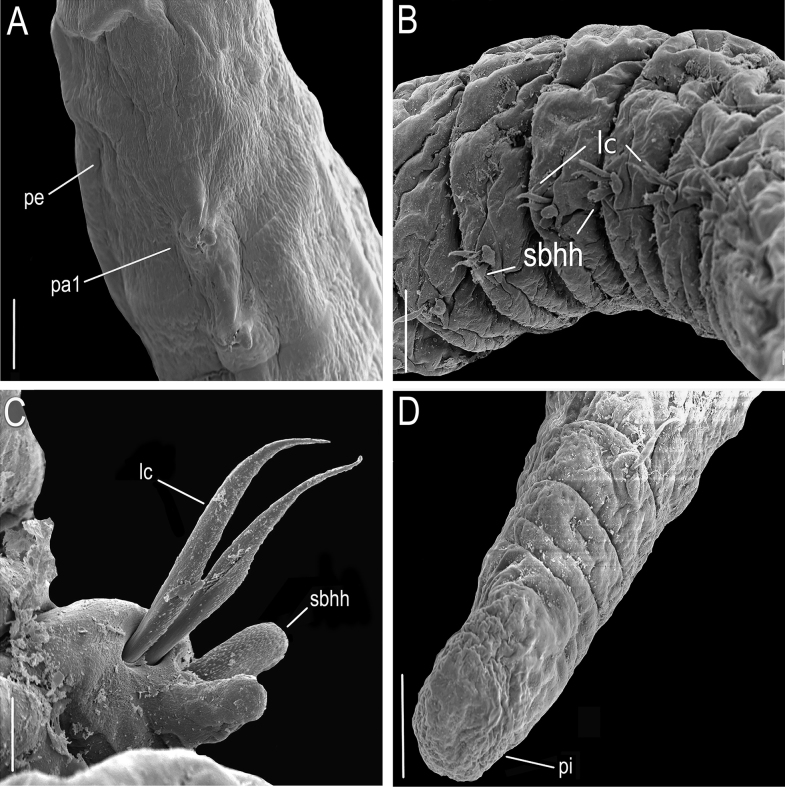
*Lumbrineriopsisdulcis* sp. nov. (SEM micrographs). (ZUEC-POL 21522) **A** parapodia 1 and 2 **B** parapodia 30–35 **C** parapodium 33 **D** pygidium, posterior end, dorsal view (chaetae are broken, except one limbate). **lc** – limbate chaetae, **pa** – parapodium, **pe** – peristome, **pi** – pygidium, **psl** – post – chaetal lobe, **sbhh** – simple bidentate hooded hook. Scale bars: 50 µm (**A, B**); 10 µm (**C**); 50 µm (**D**).

**Figure 6.**
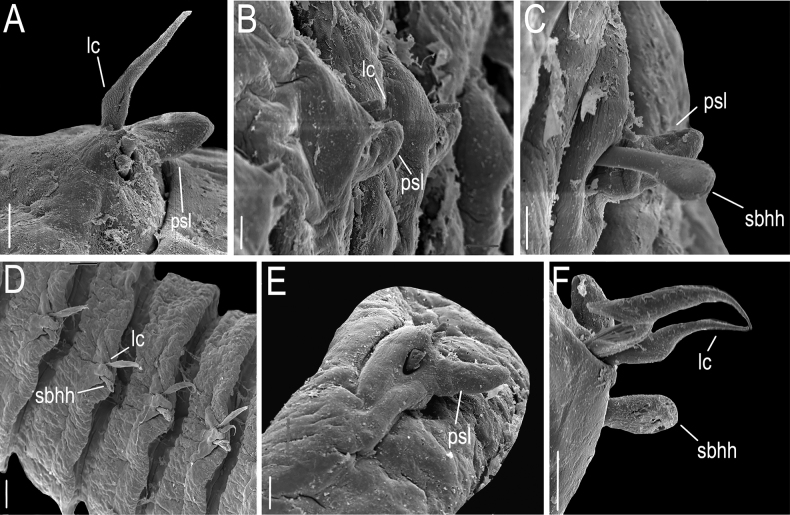
*Lumbrineriopsisdulcis* sp. nov. (SEM micrographs). (ZUEC-POL 21522) **A** parapodium 1, anterior view **B** parapodia 9 and 10, anterior view **C** parapodium 12, anterior view **D** parapodia 29 and 32 **E** parapodium 30, anterior end **F** parapodium 65, anterior view. **lc** – limbate chaetae, **psl** – post – chaetal lobe, **sbhh** – simple, bidentate, hooded hook. Scale bars: 10 µm.

**Figure 7.**
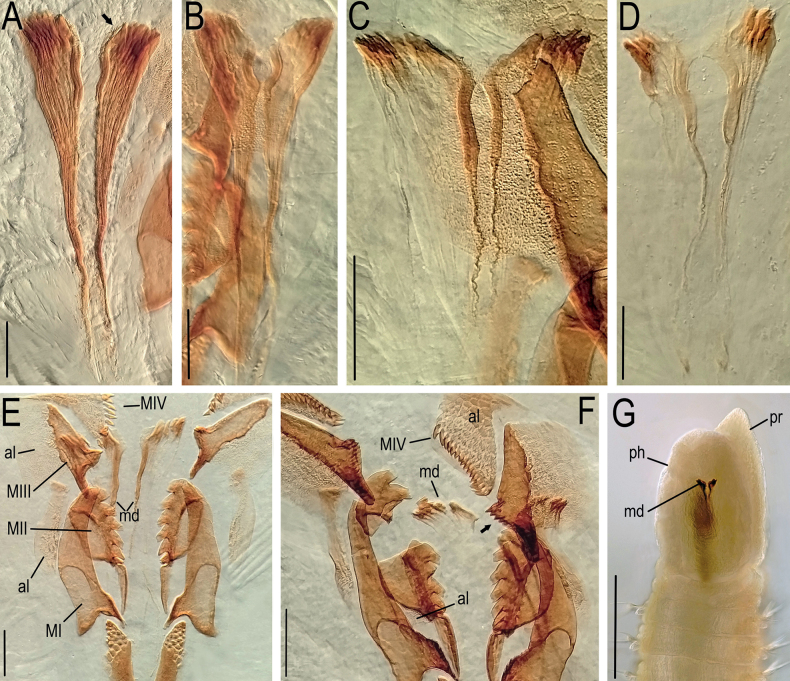
*Lumbrineriopsisdulcis* sp. nov. Mandibles (adult) **A** ZUEC-POL 22897 **B** ZUEC-POL 21526 **C** ZUEC-POL 22898 **D** ZUEC-POL 21524 jaw apparatus (adult) **E** ZUEC-POL 21525 **F** ZUEC-POL 22899 **G** ZUEC-POL 22905 anterior end, ventral view, partially everted pharynx. **al** – attachment lamella, **ph** – pharynx, **MI** – maxillae I, **MII** – maxillae II, **MIII** – maxillae III, **MIV** – maxillae IV, **md** – mandible, **pr** – prostomium. Scale bars: 45 µm (**A**); 40 µm (**B, D**); 50 µm (**C**); 48 µm (**E**); 52 µm (**F**); 200 µm (**G**).

**Figure 8.**
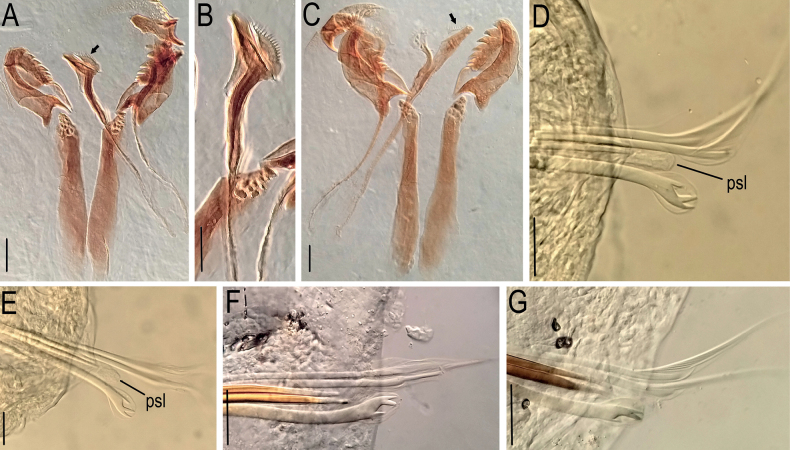
*Lumbrineriopsisdulcis* sp. nov. **A** jaw apparatus (juvenile), ZUEC-POL 22900 **B** mandible (juvenile), ZUEC-POL 22900 **C** jaw apparatus (juvenile), ZUEC-POL 22901 **D** parapodium 7, ZUEC-POL 22527 **E** parapodium 8, ZUEC-POL 22527 **F** parapodium 96, ZUEC-POL 22528 **G** parapodium 97, ZUEC-POL 22528. **psl** – post-chaetal lobe. Scale bars: 25 µm (**A–C, F, G**); 15µm (**D, E**).

Holotype complete, 0.5 mm wide, 23 mm long, 178 chaetigers. Complete adult paratypes (16 specimens): 0.3–0.75 mm wide, 4.5–43 mm long, 45–254 chaetigers. Incomplete adult paratypes (79): 0.3–0.9 mm wide, 1–71 mm long, 12–243 chaetigers. Complete juveniles paratypes (4): 0.2 mm wide, 5–11 mm long, 56–75 chaetigers. Incomplete juvenile paratypes (14): 0.1–0.25 mm wide, 5–11 mm long, 11–54 chaetigers. Body yellow or whitish in preserved specimens. Prostomium long, acuminate, 1.5–2.5 times longer than peristomium (Figs 1A, B, 4). Peristomial rings slightly demarcated, both with equal length or first ring twice as long as the second one, both slightly narrower than the following segments (Figs 1A, B, 4, 5A); without mouth pads. Jaw apparatus light brown (Fig. 7). MI forceps-like, with attachment lamella, basal external projection, basal inner portion sharper and connected to the maxillary carriers (Figs 1E, 7E). Maxillary carriers 1.5 to twice as long as MI, with tiny irregular structures in anterior end (Figs 1E, 7E). MII connected to the inner base of MI by a ligament, 6 or 7 teeth with curved and rounded end, the 2 basal teeth being the smallest (Figs 1E, F, 7E, F); attachment lamella small and not easily visible (Fig. 7F). MIII with 2 aliform expansions and 1, rarely 4 or 5 teeth (Figs 1E, G, 7E, F). Broad MIV with 11–16 denticles of the same size, tiny irregular structures in attachment lamella (Figs 1E, H, 7F). Maxillary formula: MI = 1 + 1, MII = 6 + 7, MIII = (1–5) + (1–5), MIV = (11–16) + (11–16). Mandibles composed of 2 juxtaposed rods free one to another along entire length (Fig. 7). Inner margin with a more evident longitudinal chitinous cord, ending distally in a broad plate with 10–16 denticles on its border (Figs 1C, D, 7A, 8A, B); more evident in juveniles (Figs 1D, 8A, B), sometimes not visible in adults (Fig. 7B–F). Calcareous plate and growth rings of mandibles absent. Parapodia with pre-chaetal lobe short and slightly triangular in anterior and median region (Figs 2A–C, 6A–C, 8D–G), and truncated in post-median and posterior regions (Figs 2D–F, 5C, 6D–F). Post-chaetal lobe triangular to digitiform, longer than the pre-chaetal lobe; no morphological variation along the body. Two simple limbate chaetae in the supra-acicular bundle and 1 simple bidentate hooded hook in the subacicular bundle, both present throughout the whole body (Figs 2, 3, 5C, 6A, C, F, 8D–G). Limbate chaetae with smooth edge, equal in size along the whole body, ventral ones with slightly wider limb. Hooks increasing gradually in size throughout the body; both teeth straight and directed distally, of equal size in first chaetigers (Fig. 3B, E), from chaetiger 5, inferior tooth slightly directed laterally, from chaetiger 10, inferior larger than the superior (Figs 3H, L, 8D–G). Hoods gradually shorter along the body. Parapodia with 2 transparent to light-brown aciculae (Figs 2, 3C, F, I, J, M, N, 8D–G), ventralmost projecting beyond the parapodium (Fig. 8D–G) and larger from median region (Figs 2D, 8F, G); some individuals with 3 aciculae in posterior region, about chaetiger 100 (Fig. 2F). Pygidium rounded, without anal cirri (Fig. 5D).

**Table 1.** Main morphological characters of *Lumbrineriopsis* with unfused mandibles (from the original descriptions).

Characters	*L.paradoxa* Saint-Joseph, 1888	*L.mucronata* Ehlers, 1908	*L.dulcis* sp. nov.
Color of the animal	Grey	Brownish yellow	Yellow or whitish
Prostomium shape	Elongated, rounded end	Elongated	Long, acuminate
Prostomium length	Undocumented	As long as the first 3 segments	1.5–2.5 times longer than peristomium
Peristomial rings	Well-distinguishable rings	Poorly distinguishable rings, with mouth pads, narrower than the next chaetigers	Slightly demarcated rings, without mouth pads, narrower than following chaetigers
Peristomium length	Undocumented	Slightly shorter than the following chaetigers	Slightly shorter than following chaetigers
Color of jaw apparatus	Undocumented	Dark brown	Light brown
Mandible (presence of denticles)	Present	Some large teeth	14–16 denticles
MI (shape / basal lateral projection)	Forceps / present	Forceps / absent	Forceps / present
MII (number of teeth: left + right)	5 + 9	5 + 5	6 + 7
MIII (number of teeth: left + right)	Undocumented	1 + 1	1–5 + 1–5
MIII (aliform expansions)	Undocumented	Absent	Present
MIV (number of teeth: left + right)	11 + 8	8	11–16 + 11–16
Simple limbate chaetae (number)	2 until the 21^st^ segment / 1 thereafter	4–5 anterior region / 1 thereafter	2 throughout the body
Simple bidentate hook (number)	1	1	1
Aciculae (number/color)	1 / yellow	Undocumented	2, 3 from chaetiger 100 / transparent to light brown
Type locality	Dinard, France, North Atlantic	Mouth of the Congo River, to 44 m deep, inside Foraminifera	Mouth of Doce and Paraíba do Sul rivers, to 386 m deep, soft bottoms

##### Etymology.

The specific epithet “dulcis” is a tribute to the Doce River, which flows into an estuary in the Atlantic Ocean, where *L.dulcis* is recorded.

##### Materials examined.

117 specimens, including 99 adults and 18 juveniles, from Espírito Santo (ES) and Rio de Janeiro (RJ) states. ***Holotype*: ZUEC-POL 21500** (ES), 21°4'4.76"S, 40°14'14.14"W, 150 m, 23 Jan 2012. ***Paratypes* preserved in 70% alcohol** (each lot with 1 specimen): **ZUEC-POL 21501** (ES), 19°42'26.81"S, 39°39'5.27"W, 14 m, 15 Jul 2011; **ZUEC-POL 21502** (ES), 19°49'52.15"S, 39°52'24.51"W, 30 m, 16 Jul 2011; **ZUEC-POL 21504** (ES), 20°11'25.75"S, 40°2'15.87"W, 40 m, 13 Jul 2013; **ZUEC-POL 21505** (ES), 18°52'31.35"S, 39°8'41.34"W, 40 m, 15 Jul 2013; **ZUEC-POL 21506** (ES), 20°1'3.73"S, 39°50'13.76"W, 53 m, 16 Dec 2010; **ZUEC-POL 21507** (ES), 20°12'20.26"S, 39°57'59.7"W, 50 m, 20 Jan 2012; **ZUEC-POL 21508** (ES), 19°31'51.66"S, 39°3'4.04"W, 150 m, 9 Dec 2011; **ZUEC-POL 21509** (ES), 19°36'30.6"S, 39°10'19.39"W, 400 m, 26 Jun 2013; **ZUEC-POL 21510** (ES), 21°12'14.60"S, 40°42'27.62"W, 15 m, 22 Jul 2009. ***Paratypes* on stubs for SEM: ZUEC-POL 21522** (ES), 20°34'53.42"S, 40°6'27.43"W, 50 m, 21 Jan 2012; **ZUEC-POL 21523** (ES), 20°6'44.162"S, 40°54'44.44"W, 30 m, 17 Jul 2009. ***Paratypes* mounted on slides: ZUEC-POL 21524** (ES), 19°31'51.66"S, 39°3'4.04"W, 171 m, 9 Dec 2011; **ZUEC-POL 21526** (ES), 22°8'9.28"S, 40°27'27.44"W, 103 m, 6 Mar 2009; **ZUEC-POL 22898** (ES), 19°49'57.38"S, 39°52'14.02"W, 33 m, 15 Dec 2010; **ZUEC-POL 22899** (ES), 20°11'25.75"S, 40°2'15.87"W, 40 m, 13 Jul 2013; **ZUEC-POL 22902** (ES), 19°36'30.6"S, 39°10'19.39"W, 360 m, 26 Jun 2013; **ZUEC-POL 22904** (ES), 19°49'57.38"S, 39°52'14.02"W, 33 m, 15 Dec 2010. **Additional material preserved in alcohol 70**% (each lot with 1 specimen): **ZUEC-POL 21511** (RJ), 21°40'25.12"S, 40°58'26.52"W, 18 m, 19 Jul 2009; **ZUEC-POL 21512** (RJ), 21°33'54.61"S, 40°42'53.90"W, 23 m, 20 Jul 2009; **ZUEC-POL 21513** (RJ), 21°11'0.91"S, 40°28'27.11"W, 26 m, 5 Mar 2009; **ZUEC-POL 21514** (RJ), 22°6'22.01"S, 40°43'42.32"W, 47 m, 17 Jul 2009; **ZUEC-POL 21515** (RJ), 22°6'20.06"S, 40°43'41.63"W, 47 m, 17 Jul 2009; **ZUEC-POL 21516** (RJ), 21°55'50.85"S, 40°25'59.21"W, 47 m, 23 Jul 2009; **ZUEC-POL 21517** (RJ), 22°6'41.46"S, 40°54'44.21"W. 52 m, 17 Jul 2009; **ZUEC-POL 21518** (RJ), 22°45'49.07"S, 41°45'33.35"W, 53 m, 16 Jul 2009; **ZUEC-POL 21519** (RJ), 22°8'9.28"S, 40°27'27.44"W, 65 m, 23 Feb 2009; **ZUEC-POL 21520** (RJ), 22°12'37.0"S, 40°13'18.76"W, 100 m, 24 Feb 2009; **MZUSP 4912** and **MZUSP 4913** (ES), 20°34'45.78"S, 40°11'30.74"W, 41 m, 20 Jan 2012; **MZUSP 4914** (ES), 18°52'32.61"S, 39°8'42.82"W, 40 m, 18 Jan 2012; **MZUSP 4915**, **MZUSP 4916** and **MZUSP 4917** (ES), 19°36'30.6"S, 39°10'19.39"W, 360 m, 26 Jun 2013; **MZUSP 4918** (RJ), 21°59'3.657"S, 40°25'11.070"W, 52 m, 6 Jul 2009; **MZUSP 4919** (RJ), 22°56'2.563"S, 41°53'51.338"W, 48 m, 28 Feb 2009; **MZUSP 4920** (RJ), 22°6'42.185"S, 40°54'44.182"W, 29 m, 26 Feb 2009; **MZUSP 4921** (RJ), 22°12'53.401"S, 40°51'12.488"W, 52 m, 26 Feb 2009; **MZUSP 4922** (RJ), 22°17'42.207"S, 40°26'59.691"W, 104 m, 23 Feb 2009; **MZUSP 4923** and **MZUSP 4924** (RJ), 21°44'19.481"S, 40°17'15.642"W, 50 m, 9 Mar 2009; **MZUSP 4925** (RJ), 21°44'19.481"S, 40°17'15.642"W, 49 m, 9 Mar 2009; **MZUSP 4926** (RJ), 21°44'19.591"S, 40°17'15.669"W, 50 m, 8 Jul 2009; **MZUSP 4927** (RJ), 22°37'31.715"S, 41°21'52.696"W, 54 m, 16 Jul 2009; **MZUSP 4928** (RJ), 22°11'30.609"S, 40°5'24.468"W, 44 m, 17 Jul 2009; **MZUSP 4929** (RJ), 21°10'16.281"S, 40°45'58.437"W, 21 m, 22 Jul 2009; **MZUSP 4930** (RJ), 21°44'39.982"S, 40°43'8.573"W, 21 m, 19 Jul 2009; **MZUSP 4931** (RJ), 21°39'31.643"S, 40°31'25.347"W, 28 m, 23 Jul 2009. **Additional material mounted on slides: ZUEC-POL 21525** (RJ), 22°19'3.839"S, 40°5'28.581"W, 386 m, 30 Jan 2009; **ZUEC-POL 21527** (RJ), 21°44'19.481"S, 40°17'15.642"W, 50 m, 9 Mar 2009; **ZUEC-POL 21528** (RJ), 21°28'2.517"S, 40°56'20.614"W, 16 m, 10 Mar 2009; **ZUEC-POL 22897** (RJ), 21°50'20.765"S, 40°31'38.459"W, 28 m, 13 Mar 2009; **ZUEC-POL 22900** (RJ) 22°12'37.087"S, 40°13'18.731"W, 100 m, 24 Feb 2009; **ZUEC-POL 22901** (RJ), 21°50'20.765"S, 40°31'38.459"W, 33 m, 7 Mar 2009; **ZUEC-POL 22903** (RJ), 21°50'20.765"S, 40°31'38.459"W, 28 m, 13 Mar 2009; **ZUEC-POL 22905** (RJ), 21°10'16."S, 40°45'58."W, 21 m, Nov 2020; **ZUEC-POL 22906** (RJ), 21°44'19.481"S, 40°17'15.642"W, 50 m, Nov 2020; **ZUEC-POL 21503** (RJ), 22°11'30.609"S, 40°5'24.468"W, 44 m, Nov 2020.

##### Type locality.

Southeastern Brazil, Espírito Santo state.

##### Distribution and habitat.

The distribution of this new species encompasses the states of Espírito Santo and Rio de Janeiro in southeastern Brazil, from the mouth of the Doce River to the continental slope (at depths between 14 and 400 m) in mud, sand, mixed sandy-mud bottoms, in sand with biological debris, and between limestones.

##### Remarks.

The nuchal organ was observed dorsally at the base of the prostomium in specimens analyzed under SEM (Fig. 4C) but is difficult to visualize under the stereoscopic microscope due to the small size of the specimens.

The specimens are very small, and the jaw apparatus very delicate, especially the mandibles (Figs 7A, B, E, F, 8A–C). There is no evidence of a chitinous ligament joining the pair of mandibles. In juveniles (Fig. 8A–C), each mandible is composed of a distal denticulated plate and a chitinous longitudinal rod, which remains prominent in the adult (on its inner side) and from where the mandible expands laterally and grows, which is the pattern observed in many other species of Eunicida. The color of this lateral portion can vary from intense light brown (Fig. 7A, B), colorless (Fig. 7D, E) to almost transparent (Fig. 7F), while the internal chitinous rod and anterior end remain always visible.

The notopodium, which is described as slightly developed in the description of the genus (Carrera-Parra 2006), was not observed in the new species. The post-chaetal lobe, which is digitiform, relatively short, and projected backward, sometimes is not properly positioned for visualization under the stereoscopic microscope, or sometimes appearing to be absent in slide preparation (Fig. 8F, G). Thus, care is required in positioning and placing of the coverslip to prevent some structures from being overlooked during observation under the microscope. However, the post-chaetal lobe is clearly digitiform in SEM images (Fig. 5C). There is a fixed number of two limbate chaetae along the entire body.

### ﻿Key to species of *Lumbrineriopsis* Orensanz, 1973

**Table:** 

1	Entire or partially fused mandibles	**2**
–	Unfused mandibles	**4**
2	Mandibles fused along the entire inner margin	***L.gardineri***
–	Mandibles fused up to 3/4 of the inner margin	**3**
3	MIV with 7–10 teeth on each side	***L.gasconiensis***
–	MIV with 12–13 teeth on each side	***L.tsushimaensis***
4	Four to 5 limbate chaetae on the anterior region	***L.mucronata***
–	Two limbate chaetae on the anterior region	**5**
5	Two pairs of light-brown aciculae along the body; 3 onwards from chaetiger 100	***L.dulcis***
–	Acicula single and yellow along the entire body	***L.paradoxa***

## ﻿Discussion

Of the five known species so far, two groups can be clearly observed: one group with unfused mandibles (*L.paradoxa* and *L.mucronata*) and another with fused mandibles (*L.tsushimaensis*, *L.gasconiensis*, and *L.gardineri*). The presence of unfused mandibles in Lumbrineridae, although not common, may occur as in species of the genus *Kuwaita* (Carrera-Parra 2006; Arias and Carreira-Parra 2014). Although the mandibles are not fused, both are connected by a chitinous ligament (Imagima and Higuchi 1975). Traditionally, a higher taxonomic value is given to the set of maxillae, with the mandibles supporting the definition of the species. In this study, while the maxillae were highly considered, greater attention was paid to the pair of unfused mandibles, a rare characteristic among Lumbrineridae.

*Lumbrineriopsisdulcis* sp. nov. differs markedly from *L.tsushimaensis*, *L.gasconiensis*, and *L.gardineri*, since these species have fused mandibles, with or without growth rings at the distal end. Thus, *L.dulcis* sp. nov. resembles *L.paradoxa* and *L.mucronata* (Table 1).

Although Miura (1980) deemed *L.paradoxa* and *L.mucronata* to be synonyms, the criteria adopted were few and the features superficial, while the original descriptions of Saint-Joseph (1888) and Ehlers (1908), have important information that was not considered by Miura (1980). *Lumbrineriopsisparadoxa* and *L.mucronata* differ from each other in the shape and number of teeth of MII, number of limbate chaetae along the body, and the presence of a denticulated plate in the distal portion of the mandibles of *L.paradoxa*, whereas in *L.mucronata* there is a cutting border with some larger teeth (Table 1).

Both species have been recorded in other localities (Fauvel 1914, 1923; Orensanz 1973; Camargo and Lana 1995a) but described as having partially fused mandibles. However, Saint-Joseph (1888) described *L.paradoxa* (based on a single slide-mounted specimen) as having two thin, juxtaposed rods which enlarge at the anterior end. Ehlers (1908) described *L.mucronata* (also based on a single specimen) as having a distal plate supported by “thin rods” (plural), whose cutting edge has some large teeth. Although Ehlers (1908) explained that he analyzed the mandibles laterally, the drawing clearly shows one of the “thin rods”, confirming the presence of unfused mandibles. Miura (1980) examined type material of both species, but also from localities in addition to the type localities, but he did not mention anything that resembled fused mandibles, a calcareous plate, or growth rings.

Regarding the stage of development of these two species, Saint-Joseph (1888) described a specimen 0.24 mm wide and with 93 setigers, which clearly is an adult individual, given the high number of setigers already added to the body. On the other hand, Ehlers (1908) described a complete specimen 0.5 mm wide and only 60 setigers, considered by him to be an apparent juvenile. Comparatively, it is observed that all species of the genus *Lumbrineriopsis* are very small, narrow, and long, ranging from 0.24 mm (*L.paradoxa*) (through 0.9 mm; *L.dulcis*) to 1 mm wide (*L.tsushimaensis*). Thus, although the presence of unfused mandibles is also a characteristic of young stages in Lumbrineridae, it is possible to affirm that *Lumbrineriopsis* clearly has species with fused or unfused mandibles.

Therefore, in view of this scenario, as well as the need for further clarify the validity of *L.paradoxa* and *L.mucronata*, we have followed Miura (1980) in understanding that the mandibles are not fused. However, we have considered both valid, based on the information from the original descriptions and the Worms Database (2023).

*Lumbrineriopsisdulcis* differs from *L.mucronata* in the coloration of the jaw apparatus and number of teeth of MII, MIII, and MIV, in addition to the number of limbate chaetae per parapodium (Table 1). *Lumbrineriopsisparadoxa*, on the other hand, differs from *L.dulcis* in its body coloration and the shape of MII, which, in the former is elongated and, in the latter, rounded. Both species also differ in relation to the number of teeth of MII and MIV, number of limbate chaetae in the posterior region, and number of aciculae per parapodium (Table 1).

Taxonomic studies of the genus carried out in South America by Orensanz (1973) and Camargo and Lana (1995a) have recorded the occurrence of *L.mucronata*. However, in these two studies, the mandibles are almost completely fused, with growth rings in the anterior end, indicating that the specimens may belong to another species. Camargo and Lana (1995a) also mentioned the simple bidentate hook with a basal tooth forming a 90° angle with the distal tooth, while Orensanz (1973) mentioned a double basal external projection in MI. Such characters are not present in *L.dulcis*.

Uebelacker (1984) described *L.paradoxa* with unfused mandibles from the Gulf of Mexico, with characteristics similar to *L.dulcis* and having the morphology of the juvenile mandibles, but differing from it by the absence of teeth in MIII and also bearing a greater number of limbate chaetae (2 or 3), hooks (1 or 2), and aciculae (2 or 3), in addition to the black coloration of the mandibles in juveniles, with several rows of teeth present in the anterior plate.

Considering the peculiar characteristics of *Lumbrineriopsis*, with such elongated maxillary carriers, multidentate MIV, and unfused mandibles in some species, a more in-depth analysis of its phylogenetic positioning within Lumbrineridae is necessary, since questions have already been raised (Saint Joseph 1888). Studies involving micro-CT are interesting to elucidate important details about jaw apparatus.

## Supplementary Material

XML Treatment for Lumbrineriopsis

XML Treatment for Lumbrineriopsis dulcis

## ﻿Additional information

### Author ORCIDs

Nálita Maria Scamparle Teodoro https://orcid.org/0000-0003-1901-9505

Tatiana Menchini Steiner https://orcid.org/0000-0003-4710-8927

Antonia Cecília Zacagnini Amaral https://orcid.org/0000-0002-3303-1351

### Data availability

All of the data that support the findings of this study are available in the main text.
